# Evaluation of a smartphone app to maintain skin protection behaviour in patients with work-related hand eczema as part of a maintenance programme: protocol for the quasi-randomised controlled trial ‘TecNaP-RCT’

**DOI:** 10.1186/s13063-025-09295-7

**Published:** 2025-11-26

**Authors:** Marc Rocholl, Nele Ristow, Frauke Scheufler, Kathrin Nordheider, Christoph Skudlik, Swen Malte John, Michaela Ludewig, Annika Wilke

**Affiliations:** 1 Department of Dermatology, Environmental Medicine and Health Theory, Institute for Health Research and Education, Osnabrück University, Am Finkenhügel 7a, Osnabrück, 49076 Germany; 2 Institute for Interdisciplinary Dermatological Prevention and Rehabilitation (iDerm), Osnabrück University, Am Finkenhügel 7a, Osnabrück, 49076 Germany; 3 Lower Saxony Institute of Occupational Dermatology (NIB), Osnabrück University, Am Finkenhügel 7a, Osnabrück, 49076 Germany; 4 Fachbereich Gesundheitswissenschaften, Hochschule Bochum, Gesundheitscampus 6-8, Bochum, 44801 Germany

**Keywords:** Protocol, Quasi-randomised controlled trial, Smartphone app, Work-related hand eczema, Germany

## Abstract

**Background:**

Work-related hand eczema (WRHE) is a common occupational disease in Europe, associated with impaired quality of life and a substantial socioeconomic burden. As skin protection behaviour critically influences disease course, mobile health interventions may help establish and maintain routines. The smartphone app ‘*Mein Hautschutz im Alltag*’ (MiA; German for ‘*My Skin Protection in Everyday Life*’) was developed to support patients with WRHE, but its clinical effectiveness has not yet been evaluated.

**Methods:**

This quasi-randomised controlled trial will enrol 286 adults with WRHE attending a 3-week inpatient programme at a specialised dermatology clinic. Participants are allocated in clusters based on admission date to either care as usual (control) or care as usual plus an individual goal-setting interview and a 6-month access to the MiA app (intervention). The primary outcome is change in skin protection behaviour from baseline to 6 months post-discharge. Secondary outcomes include clinical and self-rated skin condition, action control, and quality of life. Analyses will follow the intention-to-treat principle using linear mixed models.

**Discussion:**

This is the first systematic evaluation of the MiA app’s effectiveness in patients with WRHE in a quasi-randomised controlled trial. By combining a structured goal-setting interview with the MiA app, the intervention aims to support the long-term adoption and maintenance of protective behaviours in both occupational and private settings. Key limitations include the quasi-random allocation by admission date (no true randomisation), lack of allocation concealment and blinding, reliance on self-reported primary outcomes, and a 6-month follow-up. Despite these constraints, the pragmatic design aims to generate decision-relevant, real-world evidence for WRHE care.

**Trial registration:**

German Clinical Trial Registry DRKS00036627. Registered on April 14, 2025.

**Supplementary Information:**

The online version contains supplementary material available at 10.1186/s13063-025-09295-7.

## Administrative information

Note: the numbers in curly brackets in this protocol refer to SPIRIT checklist item numbers. The order of the items has been modified to group similar items (see http://www.equator-network.org/reporting-guidelines/spirit-2013-statement-defining-standard-protocol-items-for-clinical-trials/).

**Table:** 

Title {1}	Evaluation of a smartphone app to maintain skin protection behaviour in patients with work-related hand eczema as part of a maintenance programme: protocol for the quasi-randomised controlled trial ‘TecNaP-RCT’
Trial registration {2a and 2b}	This trial has been registered in the German Clinical Trials Register (DRKS) on April 14, 2025. Identifier: DRKS00036627
Protocol version {3}	Version 1.0—August 22, 2025 Version 2.0—October 30, 2025
Funding {4}	The funding of this study was provided by the Institution for Statutory Accident Insurance and Prevention in the Health and Welfare Services [*Berufsgenossenschaft für Gesundheitsdienst und Wohlfahrtspflege*] (grant number 1680).
Author details {5a}	Dr Marc Rocholl^1–3^ ^1^Department of Dermatology, Environmental Medicine and Health Theory, Institute for Health Research and Education, Osnabrück University, Am Finkenhügel 7a, 49076 Osnabrück, Germany ^2^Institute for Interdisciplinary Dermatological Prevention and Rehabilitation (iDerm) at the Osnabrück University, Osnabrück University, Am Finkenhügel 7a, 49076 Osnabrück, Germany ^3^Lower Saxony Institute of Occupational Dermatology (NIB), Osnabrück University, Am Finkenhügel 7a, 49076 Osnabrück, Germany Nele Ristow^1^ ^1^Department of Dermatology, Environmental Medicine and Health Theory, Institute for Health Research and Education, Osnabrück University, Am Finkenhügel 7a, 49076 Osnabrück, Germany Frauke Scheufler^2–3^ ^1^Department of Dermatology, Environmental Medicine and Health Theory, Institute for Health Research and Education, Osnabrück University, Am Finkenhügel 7a, 49076 Osnabrück, Germany ^2^Institute for Interdisciplinary Dermatological Prevention and Rehabilitation (iDerm) at the Osnabrück University, Osnabrück University, Am Finkenhügel 7a, 49076 Osnabrück, Germany ^3^Lower Saxony Institute of Occupational Dermatology (NIB), Osnabrück University, Am Finkenhügel 7a, 49076 Osnabrück, Germany Kathrin Nordheider^2–3^ ^1^Department of Dermatology, Environmental Medicine and Health Theory, Institute for Health Research and Education, Osnabrück University, Am Finkenhügel 7a, 49076 Osnabrück, Germany ^2^Institute for Interdisciplinary Dermatological Prevention and Rehabilitation (iDerm) at the Osnabrück University, Osnabrück University, Am Finkenhügel 7a, 49076 Osnabrück, Germany ^3^Lower Saxony Institute of Occupational Dermatology (NIB), Osnabrück University, Am Finkenhügel 7a, 49076 Osnabrück, Germany Prof. Dr Christoph Skudlik^1–3^ ^1^Department of Dermatology, Environmental Medicine and Health Theory, Institute for Health Research and Education, Osnabrück University, Am Finkenhügel 7a, 49076 Osnabrück, Germany ^2^Institute for Interdisciplinary Dermatological Prevention and Rehabilitation (iDerm) at the Osnabrück University, Osnabrück University, Am Finkenhügel 7a, 49076 Osnabrück, Germany ^3^Lower Saxony Institute of Occupational Dermatology (NIB), Osnabrück University, Am Finkenhügel 7a, 49076 Osnabrück, Germany Prof. Dr Swen Malte John^1–3^ ^1^Department of Dermatology, Environmental Medicine and Health Theory, Institute for Health Research and Education, Osnabrück University, Am Finkenhügel 7a, 49076 Osnabrück, Germany ^2^Institute for Interdisciplinary Dermatological Prevention and Rehabilitation (iDerm) at the Osnabrück University, Osnabrück University, Am Finkenhügel 7a, 49076 Osnabrück, Germany ^3^Lower Saxony Institute of Occupational Dermatology (NIB), Osnabrück University, Am Finkenhügel 7a, 49076 Osnabrück, Germany Prof. Dr Michaela Ludewig^4^ ^4^Fachbereich Gesundheitswissenschaften, Hochschule Bochum, Gesundheitscampus 6–8, 44801 Bochum, Germany Prof. Dr Annika Wilke^1–3^ ^1^Department of Dermatology, Environmental Medicine and Health Theory, Institute for Health Research and Education, Osnabrück University, Am Finkenhügel 7a, 49076 Osnabrück, Germany ^2^Institute for Interdisciplinary Dermatological Prevention and Rehabilitation (iDerm) at the Osnabrück University, Osnabrück University, Am Finkenhügel 7a, 49076 Osnabrück, Germany ^3^Lower Saxony Institute of Occupational Dermatology (NIB), Osnabrück University, Am Finkenhügel 7a, 49076 Osnabrück, Germany
Name and contact information for the trial sponsor {5b}	Prof. Dr med. Swen Malte John Am Finkenhügel 7a 49076 Osnabrück Germany Telephone: 0049 (0)541 969 2357 Fax: 0049 (0)541 969 2445 Email:johnderm@uni-osnabrueck.de
Role of sponsor {5c}	The funding institution will not be involved in the study’s conduct, collection, analysis and interpretation of the data, the writing of future manuscripts, or the decision to submit these manuscripts for publication. Furthermore, the funding institution is not involved in the preparation of this protocol.

## Introduction

### Background and rationale {6a}

The increase in smartphone technologies and mobile health (mHealth) applications presents new opportunities for the management of non-communicable diseases (NCDs). Given that NCDs are often associated with lifestyle factors such as diet, physical activity, smoking, and alcohol consumption, health behaviour is a critical determinant of treatment outcomes [1–4]. Behaviour change interventions are increasingly being implemented through app-based interventions to support the consistent adoption of health-promoting behaviours and to foster the development of long-term habits [4–6]. Against this background, the use of behaviour change apps for the prevention and management of common NCDs has been the focus of extensive research in the past. Recent meta-analyses indicate clinically relevant effects of app-based interventions on both, health-related lifestyle behaviours and clinical health outcomes. Zhou et al. [7] and Kassavou et al. [4] report significant improvements in blood pressure, medication adherence, and dietary habits, particularly for interventions that are theory-driven and personalised. In the context of obesity, Chiavarini et al. [8] and Metzendorf et al. [9] similarly demonstrate beneficial effects of digital interventions on outcomes such as waist circumference and physical activity. However, Chiavarini et al. [8] and Metzendorf et al. [9] highlight the heterogeneity of the existing evidence base and methodological limitations of the included randomised controlled trials. In summary, current findings suggest that app-based health interventions hold promise for improving health behaviours and outcomes, while underscoring the need for further methodologically rigorous and high-quality research. The underlying principles for behaviour change apps rely on the implementation of specific behaviour change techniques (BCTs), which are translated into a digital format within the app [10, 11]. Commonly utilised BCTs in such apps include goal setting, self-monitoring of behaviour, feedback on behaviour or its outcomes, and instructions on how to perform the behaviour [3, 5, 7, 12, 13]. However, the effectiveness of BCTs is context-dependent and varies according to the target health behaviour [10, 11].

Atopic dermatitis (AD) is an example of a dermatological condition in which patient behaviour can substantially influence the course and progression of the disease, depending on its severity, for instance through regular use of basic skin care, adherence to therapeutic regimens, and consistent avoidance of trigger factors [14, 15]. Behaviour change apps can support individuals with AD in monitoring event-related symptoms, such as itching, documenting therapy and adherence, and tracking the progression of the skin condition with photos [16–18]. Current research on app-based interventions for AD predominantly focusses on feasibility and user acceptance [16–18]. Some studies also indicate potential benefits of these interventions in improving health-related quality of life (HRQoL), reducing pain, and mitigating disease severity [19, 20]. However, within the field of dermatological conditions, the current evidence base remains limited, primarily due to the scarcity of efficacy studies systematically evaluating the clinical benefits of mHealth applications [19].

Work-related hand eczema (WRHE) represents another skin condition where patient behaviour plays an important role in disease management. A digital application could be of great value, but the field of WRHE has been relatively unexplored to date. WRHE is among the most frequent occupational diseases in Germany and many other European countries [21, 22], affecting various high-risk professions such as healthcare workers, hairdressers, construction workers, and metal workers, and is associated with a substantial socioeconomic burden, comprising healthcare costs, productivity loss, and work absenteeism [23–28]. In Germany, mean annual societal costs have been estimated at about €8799 per patient, with indirect costs accounting for roughly 70% of total costs [28], and additional expenses arise from retraining, rehabilitation, and pensions [22]. Furthermore, WRHE is associated with substantial consequences for affected individuals, including impaired health-related quality of life, emotional distress as well as reduced work ability [29–32]. Approximately 90% of all work-related skin diseases occur on the hands, presenting predominantly as irritant or allergic contact eczema. In addition, occupational exposures can trigger or exacerbate constitutional conditions such as AD: about 50% of individuals affected by WRHE concurrently suffer from AD [33–36]. The progression and prognosis of WRHE crucially depends on individual skin protection behaviour. Thus, outpatient and inpatient prevention programmes were developed and established in standard care which aim at empowering patients to adopt and maintain adequate skin protection, cleansing, and care measures in daily (work) life [37–40].

However, it is well established that the practical implementation of new health behaviours (e.g. skin protection behaviour) in private and occupational settings can be challenging for individuals and often constitutes a prolonged process which necessitates the development of new habits as well as effective strategies for managing barriers and relapses [41]. Health behaviour apps constitute an effective approach for providing individualised and continuous support in adopting and maintaining such behaviours [3, 5, 6]. Therefore, the smartphone application ‘MiA’ (German acronym for ‘Mein Hautschutz im Alltag’, English ‘My Skin Protection in Everyday Life’) was designed to support patients with WRHE in adopting effective skin protection behaviours and in improving self-management. The MiA app serves as a systematic maintenance programme following participation in an inpatient rehabilitation programme. It includes both informative and interactive features and offers users the opportunity to document, track, and review health-related information [42, 43]. Ristow et al. [43] piloted the MiA app and examined its user experience and adherence. However, the impact of the MiA app on the skin protection behaviour of patients with WRHE has not yet been sufficiently determined.

### Objectives {7}

Accordingly, this study aims to determine the impact of a maintenance programme—comprising an individual goal-setting interview and the use of the MiA app—on the skin protection behaviour of patients with WRHE. Skin protection behaviour in this context includes the implementation of skin protection, cleansing, and care measures in both occupational settings and private daily life. We hypothesise that the aforementioned interventions will lead to a statistically significant improvement in the adoption and maintenance of appropriate skin protection behaviour, with an expected effect size in the lower range between small and medium. Further objectives are to evaluate the effect on the skin condition of the hands, action control, HRQoL, employment status, as well as the number of days of sickness-related absence from work due to the skin condition over the follow-up period.

### Trial design {8}

The study ‘TecNaP-RCT’ is designed as a non-blinded quasi-randomised, controlled superiority trial with two parallel groups (intervention and control groups). Patients will be assigned to intervention and control groups in clusters based on their admission dates.

## Methods: participants, interventions, and outcomes

### Study setting {9}

The study will be conducted at the Institute for Interdisciplinary Dermatological Prevention and Rehabilitation at the Osnabrück University (iDerm) [44] in Osnabrück, Germany. The iDerm is an internationally recognised, interdisciplinary healthcare facility, specialising in both inpatient and outpatient individual prevention in occupational dermatology. The institute is recognised for its expertise in interprofessional care of patients with work-related skin diseases (e.g. contact dermatitis, foot eczema, and UV-induced skin cancer [45, 46]), particularly in the areas of diagnostics, therapy, patient education, and counselling.

Patients will be recruited during the 3-week complex inpatient maintenance programme, known as ‘Tertiary Individual Prevention’ (TIP), which has been described and evaluated extensively [33, 47–51]. In short, TIP consists of three phases: the first phase comprises a 3-week inpatient stay at the clinic, during which patients undergo allergy diagnostics and intensive, stage-adapted dermatological treatment. Throughout the inpatient phase, health educational and health psychology interventions (e.g. group educational sessions and individual counselling) are provided to enhance motivation and to promote modification of knowledge, attitudes, and behaviours related to appropriate skin protection, cleansing, and care [40]. Patients also practise and train the recommended skin protection measures under the supervision of occupational therapists at simulated workplace stations [52]. After the inpatient phase, a 3-week post-inpatient period follows, during which patients do not attend work and receive ongoing outpatient dermatological care within the so-called dermatologist procedure [53] to stabilise the treatment effects achieved during the inpatient stay (phase 2). In the third phase, patients return to work under optimised skin protection conditions and, if necessary, continue to receive outpatient dermatological support from their dermatologist [51].

### Eligibility criteria {10}

The following inclusion criteria must be met by patients to be eligible for the study:

- Age: >18 years (of legal age) or older
- Participation in the 3-week inpatient maintenance programme at the iDerm
- Voluntarily signed informed consent form to participate in the study
- Access to a smartphone with internet capability
- Adequate German language skills to be able to understand the app content and complete the questionnaires

Formal verification of German language proficiency is not required, as all seminars and study documents were only offered in German. Patients requiring interpreter support or who do not feel confident about participating due to the language barrier will be excluded from the study.

### Who will take informed consent? {26a}

The researchers will introduce the study and its objectives to potentially eligible participants (on site), providing a comprehensive overview of the inclusion criteria, the study design, and the processes involved in pseudonymisation. Special emphasis will be placed on data protection during the use of the MiA app. In addition to providing participant information and consent forms, visual aids will also be utilised. Participants are encouraged to ask questions about the study at any time. If the patients agree, the researchers will obtain written consent for participation. Recruitment for the study will take place at the end of the introductory seminar that patients will attend on their admission day.

### Additional consent provisions for collection and use of participant data and biological specimens {26b}

No biological samples from participants will be collected, used, or stored for ancillary studies in this trial.

## Interventions

### Explanation for the choice of comparators {6b}

This study aims to demonstrate the superiority of an app-based maintenance programme consisting of an individual goal-setting interview and the MiA app compared to no maintenance intervention. The control group will therefore receive care-as-usual (CAU), but will not receive any additional maintenance interventions during or after the inpatient maintenance programme. The control group will be given access to the app after the 6-month study period has concluded in the form of a waiting control group. Thus, all participants ultimately have access to the MiA app.

### Intervention description {11a}

Both the intervention and control groups participate in the standard inpatient individual prevention programme (CAU) at the iDerm. As described above, the programme includes a 3-week inpatient stay at the iDerm followed by a 3-week period of absence from work to facilitate skin stabilisation post-discharge. In week 4 post-discharge, participants return to work with optimised skin protection measures (e.g. optimisation of the use of protective gloves). Participants have access to outpatient dermatological care as needed, either through outpatient physicians or at the iDerm. Standard follow-up appointments, which participants may or may not be able to attend, are typically scheduled shortly before and 4 weeks after returning to work [33, 48, 51].

The intervention group additionally participates in the app-based maintenance programme, which was designed and piloted using a multi-stage, iterative, and user-centred process following the frameworks and recommendations for complex interventions [54, 55]. In this process, patients with WRHE were involved [42, 43]. A goal-setting interview is conducted face-to-face with a health educator on day 16 or 17 of the inpatient stay and lasts approximately 30 min. The interviews follow a semi-standardised protocol—while the key elements are standardised, individual needs and requirements of each patient are considered. During this session, up to five individual skin protection goals for the post-discharge period are collaboratively formulated with the participant and documented within their user profile in the app’s interface. Participants are then registered on the app and familiarised with its functionalities, followed by a test phase until discharge. The maintenance programme begins on the day of discharge (day 21) and continues for 6 months.

**Fig. 1:**
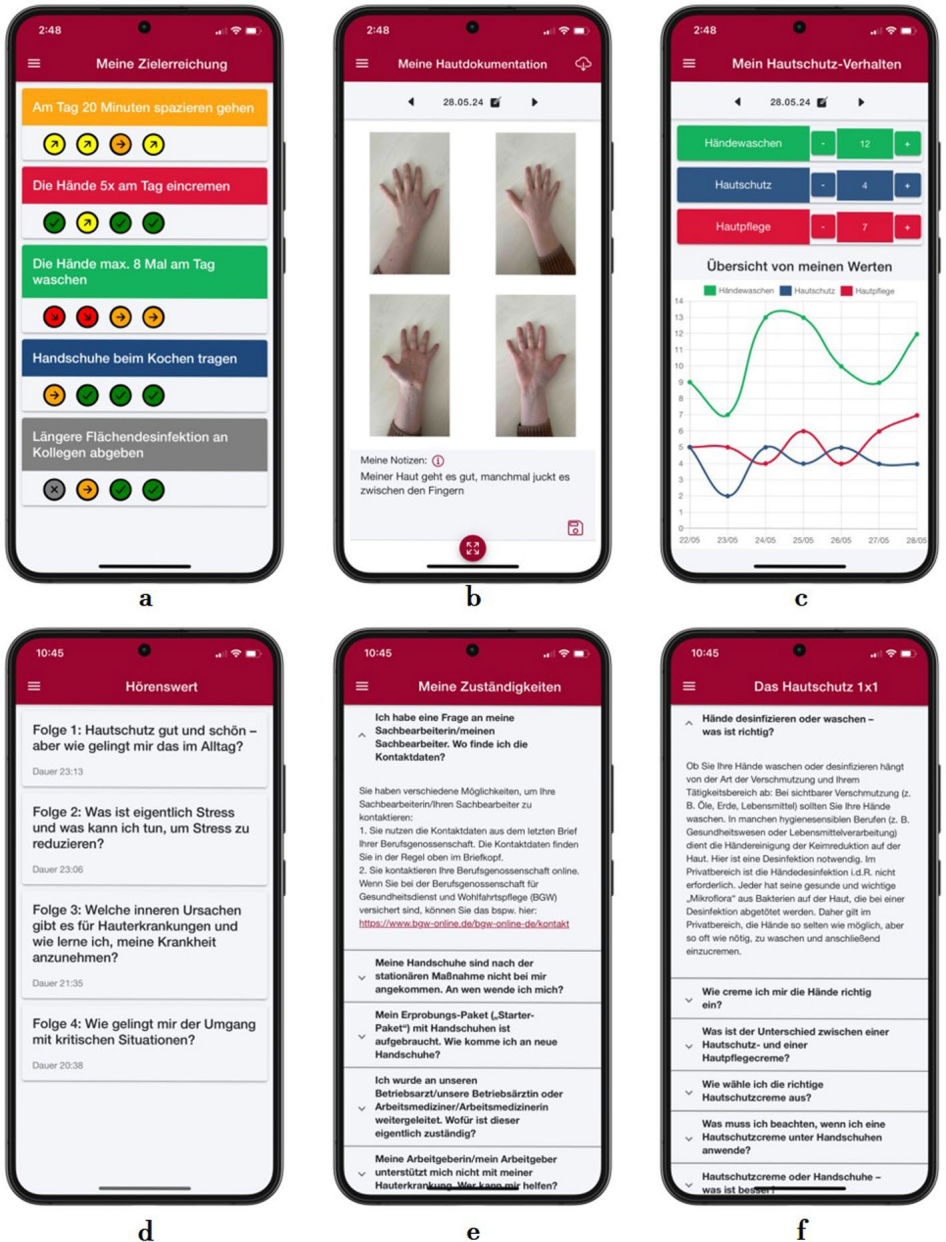
Screenshots of the MiA app. The figure displays various functions of the MiA app using screenshots. To evaluate goal achievement (screenshot **a**), participants can rate their goals on a five-point scale (red = ‘I have moved away from my goal’; orange = ‘Nothing has changed’; yellow = ‘I have come closer to my goal’; green = ‘I have achieved my goal’; grey = ‘I was unable to pursue my goal this week’). The figure also shows the functions of photographic documentation (screenshot **b**), tracking of skin protection behaviour (screenshot **c**), as well as audio content (e.g. podcasts; screenshot **d**) and text-based information (screenshots **e** and **f**)

The MiA app offers functionalities for tracking skin protection behaviour (e.g. use of skin protection and skin care creams, hand washing frequency), evaluation of goal achievement, and photographic documentation of disease course. In addition, participants have access to audio and text-based information from the patient education sessions during the inpatient stay. A detailed description of the intervention, including its components and behaviour change techniques, based on the TIDieR checklist [56] and BCT Taxonomy v1 [10], as well as first observations of user experience (UX), subjective quality, and perceived impact of the MiA app, is described by Ristow et al. [43]. Figure 1 shows screenshots to illustrate the MiA app.

### Criteria for discontinuing or modifying allocated interventions {11b}

Participants have the right to withdraw their consent to participate at any time and without giving reasons. This will not result in any disadvantages. Modification of the intervention components is neither planned nor possible.

### Strategies to improve adherence to interventions {11c}

Participants are free to use the MiA app at their discretion. There are no prescribed requirements regarding the frequency of app usage. Notably, the examination of app usage behaviour is a secondary outcome of the study. To encourage app engagement, users receive push notifications at defined intervals: a weekly reminder for goal achievement (Fridays) and photographic documentation (Sundays). Reminders for tracking skin protection behaviour are scheduled over the 6-month maintenance period as follows: a daily reminder is sent during weeks 1 and 2, weekly reminders during weeks 3 to 6, and biweekly reminders from week 7 onwards. In addition, users receive a push notification if they have not entered any information for 14 days.

App onboarding will be conducted individually with each participant after the individual goal-setting interview. Participants receive support in downloading the app and registering their personal profile. The study staff will provide tailored assistance and demonstrate the app’s functions according to each participant’s level of technical proficiency. In addition, participants will receive a flyer containing a link to a short instructional video explaining the use of the app. In case of any technical difficulties, participants may contact the research team at any time via e-mail. At the end of the 6-month usage period, participants will be informed 14 days in advance that the app will be deactivated. They will have the opportunity to download and export their data.

### Relevant concomitant care permitted or prohibited during the trial {11d}

There is no concomitant care that is prohibited during the study. Participants will receive stage-adapted dermatological care deemed necessary by their treating clinicians during and after the TIP (care-as-usual), including but not limited to emollients, topical corticosteroids, or calcineurin inhibitors as well as systemic treatment if necessary. Use of other digital health applications or educational programmes is not restricted. This procedure can be justified as follows:

WRHE comprises potentially overlapping aetiological subtypes (irritant contact dermatitis, allergic contact dermatitis, atopic dermatitis) and clinical subtypes (hyperkeratotic, acute recurrent vesicular, nummular), which often require individualised therapeutic strategies [33, 57, 58]. Restricting indicated treatments would be unethical and inconsistent with routine care; therefore, this pragmatic trial allows all necessary concomitant care, while the app and the individual goal-setting interview constitute the only study-specific interventions. Because concomitant dermatological care is neither restricted nor standardised, the secondary outcome may be influenced by co-interventions. This is inherent to our pragmatic design aimed at reflecting real-world rehabilitation. We consider this potential influence in the interpretation of results.

### Provisions for post-trial care {30}

The intervention is not anticipated to cause harm to the study participants, as a prior study [43] focussing on the UX of the MiA app indicated none. Therefore, no special post-trial care is planned. Participants in both the intervention and control groups will receive ongoing standard care within the ‘dermatologist procedure’ [53] during and after the study They may also have the opportunity to attend follow-up appointments at our clinic as part of CAU; however, participation in these appointments is not mandatory.

### Outcomes {12}

The desired primary outcome of this study is a change in the participants’ skin protection behaviour, which encompasses the implementation of skin protection measures, cleansing, and care routines in both occupational settings and private daily life. Skin protection behaviour will be measured using a 13-item instrument adapted from the scale used by Matterne et al. [59] and expanded to reflect the content of the skin protection seminars delivered within TIP. The 13 items will be aggregated into a single total score. Items are rated on a 5-point Likert scale; the total score is the mean of all items (higher values indicate better skin protection), with item 11 reverse-coded. The specific items are presented in the Supplementary information (see Additional files 1–3). The primary analysis will compare the change in the mean primary outcome score for each participant from baseline to t4 (6 months after discharge from the clinic) between groups.

The anticipated primary outcome of this study is critical, as adequate skin protection behaviour can have a significant impact on disease course and thus contribute to both the stabilisation of the skin condition and improved disease management in occupational and private settings. Furthermore, it is a commonly used outcome to evaluate the effect of patient education in trials involving patients with WRHE [37, 40, 60].

The secondary outcome measures are as follows:

- Skin condition of the hands: self-assessed using the following item: ‘How would you rate the current condition of your skin using a school grading scale?’ (1 = very good, 2 = good, 3 = satisfactory, 4 = sufficient, 5 = poor, 6 = very poor).
- Skin condition of the hands (clinical-assessed, OHSI [61, 62]): semi-quantitative scoring system for the clinical assessment of hand eczema. Range from 0 to 18. Higher values indicate a worse skin condition.
- Action control: assessed with a 9-item scale, adapted from Scholz et al. [63]; items rated on a 7-point Likert scale (1 = does not apply at all, 4 = partly applies, 7 = applies exactly). A total score is calculated as the mean of all items (higher scores indicate greater action control). Items and scoring details are provided in Additional file 2.
- HRQoL: assessed with the Dermatology Life Quality Index (DLQI) [64].
- Employment status and number of days of sickness-related absence from work (due to the skin condition) during the follow-up period. This self-reported outcome may be influenced by external factors and will be interpreted with appropriate caution. However, no relevant confounding that would impair the comparability between groups is anticipated.

Additionally, in the intervention group only, the following will be assessed:

- Impact of the MiA app on goal achievement, measured using two items scored on a four-point scale (1 = strongly agree, 2 = rather agree, 3 = rather disagree, 4 = strongly disagree). The items are presented in Additional file 3.
- Usage behaviour of the MiA app: assessed centrally via usage data collected in the web-based dashboard (so-called Cockpit) over the entire 6-month follow-up period. This includes daily values for tracking skin protection behaviour, daily values for the use of photographic documentation including the number of images (up to four), and related comments.

### Participant timeline {13}

The participant timeline of enrolment, allocation, interventions, and assessments is presented in Table 1.

**Table 1:**
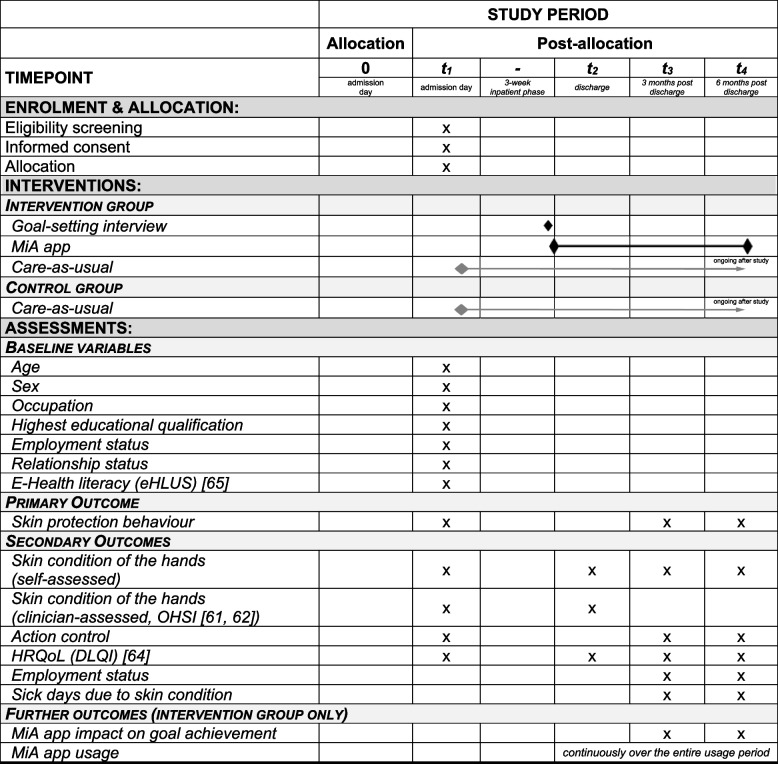
Participant timeline of enrolment, allocation, interventions, and assessments

Abbreviations: *DLQI* Dermatology Life Quality Index, *eHLUS *eHealth Literacy and Use Scale, *HRQoL *health-related quality of life, *MiA app *German acronym for ‘Mein Hautschutz im Alltag’, English ‘My Skin Protection in Everyday Life’, *OHSI* Osnabrück Hand Eczema Severity Index

### Sample size {14}

The anticipated primary outcome of the study is the improved skin protection behaviour of participants. The following assumptions and calculations form the basis for determining the total sample size: the sample size was planned a priori based on a *t*-test for independent samples, comparing the change scores (difference between t1 and t4) between the intervention and control groups. A mean effect size of *d* = 0.4 was assumed to determine the required sample size. This anticipated effect is expected to lie in the lower range between small and medium. With a significance level of *α* = 0.05 (two-tailed), a statistical power of 1 − *β* = 0.80, and an expected dropout rate of 30%, the required total sample size was calculated to be *N* = 286 participants. This total sample will be divided into the following groups: intervention group with *n* = 143 participants and control group with *n* = 143 participants. The dropout rate of 30% was based on literature reporting the effectiveness of app-based interventions for behaviour change [66, 67]. Previous studies have also indicated that the actual response rate in app-based interventions may, in some cases, exceed the projected dropout rate of 30%. The sample size calculation was conducted using G*Power software version 3.1.9.7 [68].

### Recruitment {15}

Participants in the TIP programme will be approached personally by researchers and invited to participate in the study (see item 26a). Recruitment will take place at the iDerm in Osnabrück, Germany, from May 13, 2025, until approximately October 2, 2026. Initially, only participants for the intervention group will be recruited. After a 3-week wash-out period, participants for the control group will be recruited. This procedure will then repeat: a recruitment phase for the intervention group will be followed by another 3-week wash-out phase, and then a recruitment phase for participants for the control group. The wash-out phases are needed to prevent contamination between the intervention and control groups. Throughout the recruitment phase, the recruitment status will be continuously monitored to ensure adherence to the study protocol.

## Assignment of interventions: allocation

### Sequence generation {16a}

Participants will be allocated to either the intervention or control group in clusters based on their admission date at the clinic, following a predetermined quasi-randomisation allocation scheme. The assignment to the intervention or control group is thus planned prior to the start of the study. This pragmatic approach is justified for several reasons: firstly, patients will participate in a 3-week inpatient maintenance programme. Given the duration of their stay and the potential for interpersonal interactions, it is essential to incorporate designated wash-out periods to prevent contamination between groups. Additionally, participants will be allocated in clusters rather than at the individual level. Therefore, to ensure the study is completed within an appropriate timeframe, no alternative method of random group allocation is feasible. Furthermore, to minimise potential confounding due to seasonal factors and occupational patterns, the allocation scheme ensures that participants in both groups are recruited continuously throughout the entire year. This is important because climatic influences (such as winter cold) and the seasonal admission of certain occupational groups (e.g. construction workers more often admitted in winter) may affect outcomes; thus, both groups are exposed to these factors equally.

### Concealment mechanism {16b}

Concealment of group allocation from the researchers and recruiters is not feasible, since allocation is predetermined by the date of admission to the clinic. This limitation is inherent to the design of a quasi-randomised controlled trial, where allocation is based on factors such as the date of admission and cannot be concealed from the researchers or recruiters. Potential participants are informed of their assignment to either the intervention or control group prior to providing their written informed consent.

### Implementation {16c}

The quasi-randomised allocation to the intervention and control groups was planned by two researchers (NR and ML) prior to the start of the study. The recruiters have access to these allocation documents and are not blinded to the allocation sequence.

## Assignment of interventions: blinding

### Who will be blinded {17a}

The study participants will not be blinded, as their participation in the individual goal-setting interview and the use of the app will inherently preclude blinding. Additionally, the individual goal-setting interviews will be conducted by researchers, which will also prevent blinding.

### Procedure for unblinding if needed {17b}

This study is not blinded; therefore, there is no unblinding procedure.

## Data collection and management

### Plans for assessment and collection of outcomes {18a}

All data will be collected using paper-based forms. With the exception of the OHSI [61, 62], which is a physician-administered clinical assessment tool for evaluating the severity of hand eczema, all other data will be self-reported by study participants. At baseline, the following sociodemographic data will be collected: age, sex, current occupation, highest educational qualification, employment status, and relationship status. E-Health literacy in the context of medical app usage will also be assessed using the eHealth Literacy and Use Scale (eHLUS) [65]. In addition, primary and secondary outcomes will be assessed at baseline (see ‘Outcomes {12}’). The German-language data collection forms are available from the corresponding author upon request.

### Plans to promote participant retention and complete follow-up {18b}

Participants will receive information at the beginning of the study regarding the procedures and the scope of data collection. The importance of providing complete feedback will be explicitly emphasised. The questionnaires will be sent to study participants in both groups by post, accompanied by prepaid return envelopes. In the event of no response, participants will receive a postal reminder (including a questionnaire) after 2 weeks. To enhance motivation for participation, study participants in both the intervention and control groups who return all questionnaires fully completed and in a timely manner at the four assessment points will be entered into a prize draw. Forty gift vouchers, valued at €50.00 (including VAT), are available as prizes. Eligibility for the prize draw is not influenced by the content of the responses, but only by the timely and complete return of the questionnaires. Participants are informed of this during the recruitment process.

### Data management {19}

Data collection is pseudonymised using a four-digit personal code, which is assigned to each participant in addition to their MiA code. The identification list is stored securely in a password-protected file.

Questionnaires are manually entered into IBM SPSS Statistics, version 29 (IBM Corp., Armonk, NY) [69], using a standardised codebook. Data entry is performed according to the double-entry principle. The two resulting datasets are systematically compared independently by a member of the data management team to identify and correct discrepancies. All differences are documented in order to assess the error rate. Signed consent forms are archived securely and separately from the questionnaires in locked cabinets.

### Confidentiality {27}

App access creation and data management are conducted via the web-based dashboard in which no personal data of the participants is stored. Personal identification is only possible via the separate allocation list. Data entered in the app (e.g. behaviour tracking and photos) are transmitted in encrypted form and stored on servers in Germany for 10 years. The user’s IP address changes with each login and is not stored in the app or in the dashboard. The International Mobile Equipment Identity (IMEI) is not used or stored in the app or in the dashboard.

All questionnaires are completed using paper forms. Data entry and statistical analysis are conducted by members of the in-house research team. Data will not be passed on to external parties.

The assignment list and study data are stored separately in password-protected folders. Consent forms are archived separately from the questionnaires. All data will be deleted no earlier than 10 years after study completion. Paper questionnaires will be destroyed promptly after completion of the study.

### Plans for collection, laboratory evaluation, and storage of biological specimens for genetic or molecular analysis in this trial/future use {33}

This trial will not involve the collection, laboratory evaluation, or storage of biological specimens for genetic or molecular analysis.

## Statistical methods

### Statistical methods for primary and secondary outcomes {20a}

Statistical analysis will be performed using IBM SPSS Statistics, version 29 (IBM Corp., Armonk, NY [69]). Descriptive statistical analyses will be performed to summarise patient characteristics, patient-reported outcome measures (e.g. individually assessed health, excluding the clinically assessed OHSI) and to compare groups. In the intervention arm, app usage metrics (e.g. duration and engagement) will be summarised descriptively. These analyses will include measures of central tendency (mean, median) and dispersion (range, variance, standard deviation).

For the analysis of the primary and secondary outcomes, a linear mixed model (LMM) will be used as the preferred statistical method. LMMs account for intra-group correlations (e.g. within the intervention and control groups) due to repeated measurements at time points t1–t4.

The study is designed as an efficacy trial to evaluate whether the app improves skin protection behaviour (primary outcome). Given that the app is intended as a self-management tool, the analysis will also explore its potential to support patients in independently maintaining skin health over the longer term. The LMMs will model change scores, with a pre-specific focus on the contrast between baseline (t1) and follow-up (t4). The intervention contrast will test whether the change in skin protection behaviour is significantly greater in the intervention group than in the control group. This contrast constitutes the statistical test of the intervention effect, operationalised as a group-by-time interaction.

A key advantage of LMMs is their robustness to missing data, which are common in longitudinal studies. Furthermore, LMMs allow the estimation of covariance structures even in studies with relatively small sample sizes, thereby improving the reliability of the results. All analyses will be conducted according to the intention-to-treat (ITT) principle, meaning that all randomised participants will be analysed in the groups to which they were originally assigned, regardless of their level of adherence to the intervention. This approach ensures that the effect estimates reflect the effectiveness of the intervention under real-world conditions. We acknowledge that variation in app usage in the intervention arm may attenuate the observed ITT effect; therefore, app usage will be summarised descriptively, and a pre-specified per-protocol sensitivity analysis will be conducted to assess robustness. To assess the robustness of the results, additional sensitivity analyses will be conducted, including a per-protocol (PP) analysis, which will consider only those participants who adhered to the study protocol as specified. This will include participants who complete all required assessments and, in the intervention group, use the app as specified. Comparing the results of ITT and PP analyses will enable for a more comprehensive evaluation of the stability and reliability of the findings. The significance level is set at *p* = 0.05. An overview of the intended analysis strategies is presented in Table 2.

**Table 2:** Overview of planned statistical methods

Category	Outcome	Statistical analyses methods
Primary	Skin protection behaviour	• Statistical model: linear mixed model (LMM) (change t1 to t4)
Secondary	Skin condition of the hands (self-assessed; school grades: ordinal)	• Descriptive: mean value per group and time point • Statistical model: generalised linear mixed model (GLMM)
Secondary	Action control	• Descriptive statistics: mean and standard deviation per group and measurement time • Statistical model: LMM
Secondary	Health-related quality of life	• Statistical model: LMM
Secondary	Employment status	• Descriptive; two-sample *t*-test
Secondary	Sick days due to skin condition	• Descriptive; two-sample *t*-test
Further (intervention group only)	MiA app impact on goal achievement	• Descriptive
Further (intervention group only)	MiA usage (daily values (182) per person)	• Descriptive ○ Daily usage frequency (mean, median, range, standard deviation) ○ Usage distribution by user group (frequencies, percentages) ○ Usage trends over time (time series plots, moving averages) • Correlation coefficient between measurement points ○ To analyse usage, mean scores are planned to be calculated over the intervals between t2 and t3, t2 and t4, and t3 and t4

Abbreviations: *GLMM *generalised linear mixed model, *LMM *linear mixed model

### Interim analyses {21b}

There are no interim analyses planned for this study.

### Methods for additional analyses (e.g. subgroup analyses) {20b}

No additional analyses or subgroup analyses are planned.

### Methods in analysis to handle protocol non-adherence and any statistical methods to handle missing data {20c}

The primary analysis will be performed using linear mixed models (LMM), which provide valid estimates under the assumption of missing at random (MAR). To address protocol non-adherence, the analysis will follow the intention-to-treat (ITT) principle, including all randomised participants according to their assigned groups. Additionally, sensitivity analyses will be conducted, including per-protocol (PP) analyses, to assess the impact of protocol adherence on the study outcomes.

### Plans to give access to the full protocol, participant-level data, and statistical code {31c}

The datasets used and analysed during this study will be made available by the authors upon reasonable request.

## Oversight and monitoring

### Composition of the coordinating centre and trial steering committee {5d}

This study is a single-centre trial at the iDerm in Osnabrück, Germany. The study team comprises the following roles and responsibilities: the principal investigator oversees the supervision and overall coordination of the study. The study coordinator is responsible for participant recruitment, onboarding with the MiA app, data management, and participant administration. The peer support team assists the study coordinator with recruitment and onboarding and is responsible for distributing questionnaires and reminders at timepoints t3 and t4. The study team communicates directly several times per week and convenes as needed to discuss potential protocol modifications (e.g. extension of the recruitment period) and monitor study progress. No stakeholders or external trial steering committee are involved in this study.

### Composition of the data monitoring committee, its role and reporting structure {21a}

Given that this study is not anticipated to pose any harm to the study participants and that the follow-up period of 6 months is considered rather brief, a formal data monitoring committee has not been established.

### Adverse event reporting and harms {22}

Not applicable. The research team does not anticipate any adverse effects from the intervention on the study participants.

### Frequency and plans for auditing trial conduct {23}

An audit of the trial conduct is not planned. The funding institution will receive progress reports regarding milestones and work packages.

### Plans for communicating important protocol amendments to relevant parties (e.g. trial participants, ethical committees) {25}

Minor administrative deviations from the study protocol occurring during the study will be documented, transparently reported, and justified in future trial reports. Additionally, in the case of substantive deviations, these will also be submitted to the Ethics Committee of Osnabrück University for ethical review and will be transparently reported to the funding institution and documented in the trial registry entry in the DRKS.

### Dissemination plans {31a}

The study results will be published in international peer-reviewed journals, preferably as open access publications. The publication of the final results is planned for July 2027. Additionally, the final outcomes will be presented at academic conferences organised by national or international research networks or professional associations, such as the Working Group for Occupational and Environmental Dermatology [in German: *Arbeitsgemeinschaft für Berufs- und Umweltdermatologie*]. There are no publication restrictions imposed by the study sponsor or the company that developed the MiA app. All findings, including statistically significant and non-significant results, as well as any reports of adverse effects, will be made publicly available.

## Discussion

This study protocol describes a quasi-randomised controlled intervention trial designed to determine the impact of an app-based maintenance programme consisting of an individual goal-setting interview and the MiA app on skin protection behaviour in patients with WRHE.

Advances in digitalisation of healthcare in recent years has expanded opportunities for patient care and led to the development of innovative interventions and applications, such as smartphone apps designed to promote self-management in lifestyle-related and chronic conditions [70, 71]. The potential of smartphone apps has been widely described across various settings, particularly with respect to self-management or to support health behaviour change [3, 72–74]. However, in the field of occupational dermatology, there is currently no digital solution specifically tailored to the needs of patients with WRHE. While previous studies describe the systematic development [42] and piloting of the MiA app [43], the present protocol outlines the first study designed to evaluate its clinical effectiveness within a quasi-randomised controlled trial for the first time. Within occupational dermatology, the MiA app constitutes a novel and innovative approach that may offer several advantages for patients. Assuming its effectiveness, the app can provide users—beyond participation in the TIP programme—with access to evidence-based information, prepared for the target population in multiple formats (e.g. written and audio). Notably, the iterative and participatory development process described by Ristow et al. [42], which followed established frameworks and recommendations for complex interventions [54, 55], represents a key strength of the MiA app. The app may support both the initiation and maintenance of new health behaviours. In particular, the ability for patients to set individual goals during the goal-setting interview, as well as to monitor their achievement, stands out as a unique feature of the intervention. Goal-setting and monitoring goal achievement are frequently used BCTs in smartphone apps. Evidence suggests that the ability to set goals is an important element in behaviour change apps [3, 5, 12, 13, 75].

### Limitations

Several limitations of the intervention study described in this protocol should be acknowledged.

#### Lack of blinding

First, the absence of blinding increases the potential for bias. Performance bias may arise if knowledge of group allocation—consciously or unconsciously—leads to differences in concomitant medical care or, in our case in particular, patient education and counselling during TIP. Detection (observer) bias in our study may arise as the dermatologists who assess skin condition (e.g. OHSI at t1 or prior to discharge at t2) are aware of participants’ allocation. Taken together, these limitations increase the risk of biased inference and misinterpretation of the findings [76–78]—as may also be the case in our study. Although blinding is an important aspect of conducting clinical trials, it is not always feasible. Notably, a lack of blinding is common in studies of app-based interventions (e.g. [79–81]); indeed, blinding of participants and providers is inherently infeasible in app-supported behavioural interventions [82, 83]. In this study, furthermore, the person responsible for statistical analyses is also involved in participant recruitment and in conducting the individual goal-setting interviews; therefore, blinding of the data analysis is not feasible. To mitigate the outlined concerns, we plan for the statistical analyses to undergo independent review by an experienced statistician upon study completion and prior to final reporting.

#### Outcomes

The primary outcome of this intervention study is the participants’ self-reported skin protection behaviour. Self-reported outcomes are appropriate—and often indispensable—when no equivalent objective outcome is available. Although objective clinical measures exist in (occupational) dermatology, the assessment of patients’ behaviour typically relies on self-report; direct field observations would be methodologically desirable but are rarely feasible due to logistical and resource constraints. However, self-reports are susceptible to interpretation and reporting biases (e.g. social desirability), which may introduce measurement error and inflate estimated intervention effects. The absence of blinding (as discussed above) may further exacerbate these risks; thus, any observed effects cannot rule out expectancy (placebo) effects or related Hawthorne effect [83–86].

While clinical outcomes, such as hand eczema severity measured by the OHSI, are usually considered as the gold standard, this approach is also not feasible for the assessment points t3 and t4 in this study, as multiple in-person follow-up visits at the clinic in Osnabrück would not be practical due to the nationwide distribution of patients across Germany. This also applies to the use of objective measures such as skin barrier function tests as the primary endpoint, which requires specialised equipment typically available only in specialised centres, together with inter-site device calibration and staff training to ensure standardised measurements. Moreover, conducting outpatient clinical assessments of skin condition via the OHSI would pose challenges regarding reliability and objectivity if performed by various outpatient dermatologists without specific training in this measure. While Weigandt et al. [20] demonstrated that photo-based assessments of eczema severity correlate closely with in-person dermatologist ratings, teledermatology may facilitate remote follow-up; however, previous experience at our centre has encountered challenges with insufficient image quality and the inability to perform a reliable physical examination [87]. Given these limitations in assessing skin condition remotely, we decided against implementing remote assessments at t3 and t4. Against this background, we decided to combine the assessment of disease severity by dermatologists using the OHSI at t1 and t2 with patients’ self-assessments based on the school grading scale at t1, t2, t3, and t4. Among different single-item global rating scales, none has demonstrated clear superiority in terms of correlation with established clinical gold standards [88]. They may nonetheless be considered appropriate, as easy-to-use instruments, particularly in large observational or intervention studies where hand eczema needs to be assessed in larger cohorts at multiple points in time, as is the case in TecNaP-RCT [88].

#### Quasi-randomisation

In this trial, allocation to the intervention and control groups is conducted in clusters based on the date of admission to the clinic. This constitutes a pragmatic, quasi-randomised approach rather than strict randomisation and, as such, carries a risk of bias. In particular, confounding may influence any observed effects (i.e. differences in outcomes could be attributable to factors other than the intervention) [86]. Moreover, quasi-randomisation and the absence of allocation concealment render the study susceptible to allocation and selection bias. Knowledge of forthcoming group assignment may lead to systematic differences between groups and, in turn, affect study outcomes [86, 89]. Accordingly, we plan adjustment analyses (e.g. in the event of baseline imbalances) and will conduct sensitivity analyses to test the robustness of the results.

Notwithstanding these limitations, the chosen allocation method is a pragmatic alternative that enables the generation of decision-relevant evidence for the care of patients with WRHE. Quasi-experimental designs are well suited to producing causal inferences that are applicable to real-world implementation [90]. This is particularly pertinent here, as the TIP inpatient rehabilitation programme operates within relatively fixed parameters (treatment, patient education and counselling workflows) that align more naturally with a pragmatic study design than with an artificial experimental setting—an approach that could otherwise threaten external validity. In addition to the planned adjustment and sensitivity analyses, we will report the study in accordance with the updated guideline for reporting randomised trials (CONSORT 2025 Statement [91]) with particular attention to the CONSORT extension for cluster trials [92].

#### Follow-up period and generalisability

Finally, a 6-month follow-up period can be regarded as relatively short, although follow-up periods of this length are common in studies assessing the effectiveness of app-based interventions (e.g. [7, 93–95]). While longer follow-up would be valuable, evidence from other app-based studies indicates a substantial decrease in engagement over time. Moreover, longer duration does not necessarily yield stronger effects; in digital interventions, benefits may be most pronounced early on [7, 17, 67, 96]. The choice of a 6-month follow-up period was extensively discussed within the research team. Given the high dropout rates and decreasing app usage reported in similar studies, a 6-month period was deemed appropriate.

From a health psychology perspective, 6 months can also be considered a reasonable period for the establishment of new habits, as research indicates that habit formation typically requires between 2 and 5 months for health-related behaviours to become automatic and ingrained, with individual variability in timeframes depending on behaviour complexity and context [97–99]. However, longer follow-up periods may be beneficial to fully capture the sustainability of behaviour change and relapse prevention, which should therefore be acknowledged as a limitation of this study and potential future research direction.

Finally, we acknowledge that this is a single-centre study conducted in Germany; consequently, the generalisability of the findings may be limited with respect to both other skin conditions and healthcare settings in other countries.

### Future directions

In summary, whereas Ristow et al. [43] focussed on UX, subjective quality, and perceived impact of the MiA app on goal achievement, this study will provide insights into the effects of the individual goal-setting interview and the MiA app on skin protection behaviour in patients with WRHE. Despite the limitations described above, the results of this study are expected to contribute to improved care for patients with WRHE in Germany in the medium term. In the long term, the app-based intervention could potentially be adapted for use in other European countries facing similar challenges with regard to WRHE.

## Trial status

The current version of the protocol is version 2.0, dated October 30, 2025. Version 1.0 was submitted on August 22, 2025. Patient recruitment began on May 13, 2025, and is estimated to be completed in October 2026.

## Supplementary Information

Additional file 1. Items used to measure the primary outcome (‘skin protection behaviour’), with original wording in German and the corresponding translation in English

Additional file 2. Items used to measure the secondary outcome (‘action control’), with original wording in German and the corresponding translation in English

Additional file 3. Items used to measure the secondary outcome (‘impact on goal achievement’), with original wording in German and the corresponding translation in English

## Data Availability

All investigators will have access to the full trial datasets; there will be no restrictions on access. The datasets used and analysed during this study will be made available from the authors upon reasonable request.

## Abbreviations

AD: Atopic dermatitis
BCTs: Behaviour change techniques
CAU: Care-as-usual
CONSORT: Consolidated Standards of Reporting Trials
DLQI: Dermatology Life Quality Index
DRKS: German acronym for ‘*Deutsches Register Klinischer Studien*’, English ‘German Clinical Trials Register’
eHLUS: EHealth Literacy and Use Scale
GLMM: Generalised linear mixed model
HRQoL: Health-related quality of life
iDerm: Institute for Interdisciplinary Dermatological Prevention and Rehabilitation at Osnabrück University
IMEI: International Mobile Equipment Identity
ITT: Intention-to-treat
LMM: Linear mixed model
MAR: Missing at random
mHealth: Mobile health
MiA: German acronym for ‘*Mein Hautschutz im Alltag*’, English ‘My Skin Protection in Everyday Life’
NCDs: Non-communicable diseases
OHSI: Osnabrück Hand Eczema Severity Index
PP: Per-protocol
TIP: Tertiary Individual Prevention
UX: User experience
WRHE: Work-related hand eczema
