# Supplementary material for: Evaluation of a smartphone app to maintain skin protection behaviour in patients with work-related hand eczema as part of a maintenance programme: protocol for the quasi-randomised controlled trial ‘TecNaP-RCT’
Source: Trials. 2025 Nov 26;26:557. doi: 10.1186/s13063-025-09295-7 (PMC12670733; doi:10.1186/s13063-025-09295-7)
Supplement: Supplementary file 1 — Additional file 1. Items used to measure the primary outcome (‘skin protection behaviour’), with original wording in German and the corresponding translation in English [file 13063_2025_9295_MOESM1_ESM.docx]

**Additional Information – Study protocol ‘TecNaP-RCT’**

**Additional File 1**

**Title: Items used to measure the primary outcome (‘skin protection behaviour’), with original wording in German and the corresponding translation in English.**

| **No.** | **Item in German** | **Item in English** |
| --- | --- | --- |
| 1 | Ich verwende regelmäßig Handcreme bei der Arbeit | I regularly use hand cream at work. |
| 2 | Ich verwende regelmäßig Handcreme in der Freizeit | I regularly use hand cream during leisure time. |
| 3 | Ich verwende bei Bedarf (z. B. trockene Haut) über Nacht Handcreme (z. B. mit darüber gezogenen Baumwollhandschuhen) | When needed (e.g., in case of dry skin), I apply hand cream overnight (e.g., with cotton gloves worn on top). |
| 4 | Ich verwende regelmäßig Schutzhandschuhe bei der Arbeit | I regularly wear protective gloves at work. |
| 5 | Ich verwende regelmäßig Schutzhandschuhe in der Freizeit (z. B. im Haushalt oder bei Gartenarbeit) | I regularly wear protective gloves during leisure time (e.g., when doing housework or gardening). |
| 6 | Bei der Reinigung meiner Hände verwende ich, so oft es geht, hautschonende Reinigungsmittel | When cleaning my hands, I use skin-friendly cleaning agents as often as possible. |
| 7 | Ich vermeide in der Regel den Hautkontakt zu reizenden Substanzen | I generally avoid skin contact with irritating substances. |
| 8 | Ich verwende am Arbeitsplatz ganz bewusst bestimmte Handschuhe, um mich bei verschiedenen hautbelastenden Tätigkeiten zu schützen | At the workplace, I deliberately use specific gloves to protect myself during various skin-stressing tasks. |
| 9 | Ich verwende in der Freizeit ganz bewusst bestimmte Handschuhe, um mich bei verschiedenen hautbelastenden Tätigkeiten zu schützen | In my leisure time, I deliberately use specific gloves to protect myself during various skin-stressing tasks. |
| 10 | Ich reinige meine Hände nur dann, wenn sie sichtbar verschmutzt sind | I only clean my hands when they are visibly soiled. |
| 11 | Um die Reinigungswirkung beim Händewaschen zu verstärken, verwende ich regelmäßig Bürsten, Bimssteine oder Reibemittel (z. B. Handwaschpaste mit Sand) | To increase the cleaning effect, I regularly use brushes, pumice stones, or abrasive agents (e.g., hand washing paste with sand) when washing my hands. |
| 12 | Bei der Reinigung meiner Hände verwende ich in der Regel lauwarmes Wasser | When cleaning my hands, I usually use lukewarm water. |
| 13 | Ich trockne meine Hände in der Regel vorsichtig ab („trocken tupfen“) und achte darauf, dass dabei wenig Reibung entsteht | I usually dry my hands carefully (‘pat them dry’) and make sure to minimize friction in the process. |
| **Scale**: 5-point scale (fully applies; rather applies; applies partially; rather does not apply; does not apply at all). | | |
