# Supplementary material for: Evaluation of a smartphone app to maintain skin protection behaviour in patients with work-related hand eczema as part of a maintenance programme: protocol for the quasi-randomised controlled trial ‘TecNaP-RCT’
Source: Trials. 2025 Nov 26;26:557. doi: 10.1186/s13063-025-09295-7 (PMC12670733; doi:10.1186/s13063-025-09295-7)
Supplement: Supplementary file 2 — Additional file 2. Items used to measure the secondary outcome (‘action control’), with original wording in German and the corresponding translation in English [file 13063_2025_9295_MOESM2_ESM.docx]

**Additional Information – Study protocol ‘TecNaP-RCT’**

**Additional File 2**

**Title:** **Items used to measure the secondary outcome (‘action control’), with original wording in German and the corresponding translation in English.**

| **No.** | **Item in German** | **Item in English** |
| --- | --- | --- |
|  | **In den letzten 7 Tagen habe ich …** | **In the past 7 days, I have …** |
| 1 | … mich stets beobachtet, ob ich Hautschutzmaßnahmen so umsetze, wie ich mir das vorgenommen habe. | … continuously monitored myself to ensure I am implementing skin protection measures as I intended to. |
| 2 | … genau darauf geachtet, auch wirklich regelmäßig Hautschutzmaßnahmen umzusetzen. | … paid close attention to actually implementing skin protection measures on a regular basis. |
| 3 | … mich beobachtet, ob ich Hautschutzmaßnahmen so intensiv umsetze, wie ich mir das vorgenommen habe. | … monitored myself to see whether I am implementing skin protection measures as thoroughly as intended. |
| 4 | … mir meine Vorsätze für meine Hautschutzmaßnahmen stets vor Augen gehalten. | … constantly kept my intentions regarding skin protection measures in mind. |
| 5 | … sehr oft an meine Vorsätze in Bezug auf meine persönlichen Hautschutzmaßnahmen gedacht. | … frequently thought about my intentions concerning my personal skin protection measures. |
| 6 | … meine Vorsätze für meine Hautschutzmaßnahmen ständig im Hinterkopf gehabt. | … continually kept my intentions for my skin protection measures in mind. |
| 7 | … mich sehr bemüht, wirklich regelmäßig Hautschutzmaßnahmen umzusetzen. | … made a strong effort to actually implement skin protection measures on a regular basis. |
| 8 | … alles getan, um so oft Hautschutzmaßnahmen umzusetzen, wie ich es mir vorgenommen habe. | … did everything I could to implement skin protection measures as often as I intended. |
| 9 | … sehr versucht, meinen Vorsätzen für meine persönlichen Hautschutzmaßnahmen nachzukommen. | … made a great effort to follow through with my intentions for personal skin protection measures. |
| **Scale**: 7-point scale (1 = does not apply; 4 = partly applies; 7 = fully applies). | | |

**Comment:**

The original scale by Scholz et al.^[[1]](#footnote-1)^ was adapted for use with the target behaviour in this study. The scale comprises of three subfacets self-monitoring, awareness of standards, and self-regulatory effort, each assessed with three items. The item steam ‘*In the last 7 days I have ...*’ precedes each item; for example ‘continuously monitored myself to ensure I am implementing skin protection measures as I intended to’ (self-monitoring), ‘continually kept my intentions for my skin protection measures in mind’ (awareness of standards) or ‘made a strong effort to actually implement skin protection measures on a regular basis’ (self-regulatory effort). *Scoring:* Primary endpoint is the total action-control score (mean of all 9 items; higher scores indicate greater action control). Subfacet means (3 items each) may be reported descriptively. Prior work has shown that the three subfacets load on a single action-control factor with high internal consistency.

1. Scholz U, Keller R, Perren S. Predicting behavioral intentions and physical exercise: a test of the health action process approach at the intrapersonal level. Health Psychol. 2009;28:702–8. doi:10.1037/a0016088. [↑](#footnote-ref-1)
