# Supplementary material for: Evaluation of a smartphone app to maintain skin protection behaviour in patients with work-related hand eczema as part of a maintenance programme: protocol for the quasi-randomised controlled trial ‘TecNaP-RCT’
Source: Trials. 2025 Nov 26;26:557. doi: 10.1186/s13063-025-09295-7 (PMC12670733; doi:10.1186/s13063-025-09295-7)
Supplement: Supplementary file 3 — Additional file 3. Items used to measure the secondary outcome (‘impact on goal achievement’), with original wording in German and the corresponding translation in English [file 13063_2025_9295_MOESM3_ESM.docx]

**Additional Information – Study protocol ‘TecNaP-RCT’**

**Additional File 3**

**Title: Items used to measure the secondary outcome (‘impact on goal achievement’), with original wording in German and the corresponding translation in English.**

| **No.** | **Item in German** | **Item in English** |
| --- | --- | --- |
| 1 | Die MiA-App hat mir geholfen, meine persönlichen Ziele nicht aus den Augen zu verlieren. | The MiA app helped me to stay focused on my personal goals. |
| 2 | Die MiA-App hat mir geholfen, meine persönlichen Ziele zu erreichen. | The MiA app helped me to achieve my personal goals. |
| **Scale**: 4-point scale (fully agree; rather agree; rather disagree; fully disagree). | | |
